# A systematic review of communication interventions to help healthcare professionals discuss genetic testing for breast cancer

**DOI:** 10.1007/s10549-020-05741-z

**Published:** 2020-06-23

**Authors:** Rachel Starkings, Valerie Shilling, Valerie Jenkins, Lesley Fallowfield

**Affiliations:** SHORE-C, Brighton and Sussex Medical School, Brighton, BN1 9RX England

**Keywords:** Breast cancer, Communication, Education, Genetic counseling, Genetic testing, Systematic review

## Abstract

**Purpose:**

This systematic review examined educational training interventions for healthcare professionals (HCPs) discussing genetic testing and risk for hereditary breast cancer. There was a particular focus on the presence, and content, of communication elements within these packages.

**Methods:**

Searches were run via CINAHL, EMBASE, PUBMED, and PsychInfo in February 2019 to identify training interventions available to HCPs with reference to communication skills. Studies were assessed for quality, with relevant intervention and outcome data extracted and synthesized. This review followed the Preferred Reporting Items for Systematic Review and Meta-analyses (PRISMA) statement and was registered on the PROSPERO database (CRD42019124010).

**Results:**

Of 3,988 items, seven papers, two of which were linked, were eligible for inclusion. There was a mix of randomized and single arm studies with web-based and face-to-face interventions. Content included an overview of genetics, hereditary and familial background, and recommended practice techniques. Outcomes focused on communication, self-efficacy, knowledge, and satisfaction. Interventions were designed for genetic counselors, physicians, primary care physicians (PCPs), medical students, and nurses. None of the papers featured oncologists or surgeons.

**Conclusions:**

This review revealed an overall lack of publications which evaluated interventions to assist HCPs discussing hereditary breast cancer risk and testing. Studies failed to operationalize which ‘communication skills’ they included, nor did they consistently report randomization, outcome measures, or analysis.

Discussing the need for, and management of, genetic testing for inherited cancer risk with individuals and their families can be challenging. As genetic testing in breast cancer becomes more common, the provision of specific communication-based training programs, with reference to genetic testing, risk assessments, and counseling skills is warranted.

**Electronic supplementary material:**

The online version of this article (10.1007/s10549-020-05741-z) contains supplementary material, which is available to authorized users.

## Introduction

A generally greater uptake of cancer risk assessments, availability of direct-to-consumer tests, and growing insurance coverage for these services has led to increasing demand for genetic services [1, 2]. This is particularly evident regarding hereditary breast cancer, which reportedly comprises ≈5% of all breast cancer presentations, and where BRCA1 and 2 mutations put an individual at higher individual risk of additional cancers [3–6].

The call for accessible genetic services has grown exponentially since the identification of BRCA1 (1994) and BRCA2 genes (1995). Furthermore, publicity surrounding high-profile celebrity cases (e.g., Angelina Jolie) significantly increased public awareness of the genetic risk to cancer [3, 7–9]. However, such demand generally exceeds the availability of counseling services [9–11]. Consequently, many different healthcare providers (HCPs) may be involved in discussions about the need for genetic testing, the consequences of a test result for an individual, and the ensuing implications for other family members [10]. Genetic counselors or geneticists, primary care physicians making genetic referrals, specialist breast care nurses, oncologists, or surgeons may all have a significant role in explaining the issues surrounding testing with individuals and their families [3, 5, 8, 12, 13]. The timing and format of these conversations can impact the value they have for individuals and the overall health system [4]. For example, a primary care physician conducting an early risk assessment appropriately for someone worried about their own risk might well be sufficient and ease the pressure on genetic services [3, 14].

Providing genetic risk assessment, testing, and counseling necessitates an appropriate knowledge base and good communication skills [15] including the ability to build rapport, while providing a clear explanation of risk, empathy, and a genuinely client-centered approach [16–18]. Rather than being prescriptive, the counselor needs to provide guidance and support thereby reinforcing an individual’s autonomy [19].

Genetic counseling has historically consisted of two main components, a pre- and post-test discussion. The pre-test session outlines information about the test and its outcomes, the provision of informed consent, and a discussion about family history and risk assessment [20–22]. If the patient proceeds with testing, the post-test session builds on pre-test information while providing the test result itself [20]. Some patients may decline testing or there may not be a clinical need to proceed. Other methods of counseling are being used to provide services for a growing population with geographical diversity, such as telephonic sessions, group introductions, and pre- and post-test sessions delivered by different health professionals (e.g., a genetic counselor only seeing high-risk patients for a post-test consultation) [23].

Individual responses to test results vary, be they positive, negative, or a variant of uncertain significance (VUS). Effective counseling should then pre-empt and prepare a counselee for each of these outcomes with appropriate psychosocial support [17, 21]. In order to do this, HCPs need commensurate levels of genetic knowledge and interpersonal skills to help an individual navigate complex ethical, familial, and legal issues [5, 24]. Not only do individuals have varying information needs, but HCPs need to be able to interpret and clearly convey the risk information, and corresponding referral and management options [19, 20, 25, 26]. Consultations that are vague, overly complex, jargonistic, or are dominated by the clinician are deemed the most unsatisfactory [19, 26, 27]. Both members of the public and HCPs may have poor numeracy and struggle to understand risk-based information [19, 28]. This confusion can prohibit engagement and lead to conversations that result in misinformed decision making [15, 26]. Communication should then be both process and content-focused, providing correct information with relevant interpersonal skills.

These conversations play an important role in informing not only an individual’s own choices about treatment and surveillance, but potentially those of other members of their family [4, 7, 29–31]. Unfortunately, there is evidence that HCPs without specific genetic training often lack confidence and knowledge about the referral pathway, the genetic background of inheritance, or how to speak to individuals about genetic testing and risk assessment [11, 24, 32–34]. There are also concerns that without appropriate guidance, individuals may struggle to manage the psychological burden of testing and the future implications of any results [4, 35].

There has been considerable research around aids and interventions for the counselee [5, 24, 30, 36], along with exploration of the genetic counseling process and dialogue [18, 37]. However, training interventions directed at HCPs communicating risk and genetic based information are rare and less well evaluated.

Our systematic review examined the published literature to identify if, and how, communication skills were being included in educational materials for HCPs discussing hereditary breast cancer. As noted, good communication involves both process and content-based work. Reference to ‘communication skills’ within this paper then refers to a combination of both. Understanding this landscape can inform future training programs as a broader group of HCPs become involved in conversations about genetic testing for hereditary mutations relevant to breast cancer risk.

## Methods

The review is registered on the PROSPERO database (CRD42019124010) and was conducted following the Preferred Reporting Items for Systematic Review and Meta-analyses (PRISMA) statement.

Our specific objectives were to (a) identify published educational interventions available for HCPs discussing genetic testing in relation to breast cancer risk, (b) understand the components that make up these programs, including how communication skills were operationalized/defined, and (c) synthesize reported outcomes of intervention efficacy as dictated by the programs themselves. We consider ‘HCP’ to include genetic counselors/geneticists, medical students, nurses, oncologists, primary care providers (PCPs), and surgeons. This array of disciplines allows for the evolving model of HCPs who might conduct genetic conversations. We did not specify a control arm as the emphasis for this review was to understand the current landscape of available materials. We anticipated studies to report on outcomes such as HCP confidence and competence along with including their own working model or definition of communication skills.

### Eligibility

Studies were included if they were published between 1995 and February 2019, with the earlier parameter selected in line with the discovery of the BRCA genes. We anticipated a small and disparate body of literature and as such were deliberately inclusive in our criteria to maximize the number of eligible studies identified; papers were eligible if they reported an educational intervention conveying genetic information. These studies did not have to report solely on testing within breast cancer, as they could be embedded within a larger suite of training. However, they did need to directly reference breast cancer genetic material. We also included papers discussing the development of these interventions.

We excluded studies that purely reported population-based testing or outcomes, including patient or public understanding of genetic testing, and those that only discussed analytic components of an assay or clinical outcomes associated with genetic test results, e.g., chemotherapy outcomes. Finally, we omitted papers that aimed to educate the public or patients on genetic testing, did not include any information relevant to hereditary breast cancer, were not available in English, or were conference abstracts/letters/editorials.

### Procedure

Our search terms were grouped into four categories for healthcare professionals, communication, interventions, and the population (Supplement 1).

Terms were combined with Boolean operators and searched using EMBASE, CINAHL, PUBMED, and PsycINFO. Searches were saved into an EndNote X7 library and duplicates were removed using the in-built feature, coupled with a hand search.

**Fig. 1:**
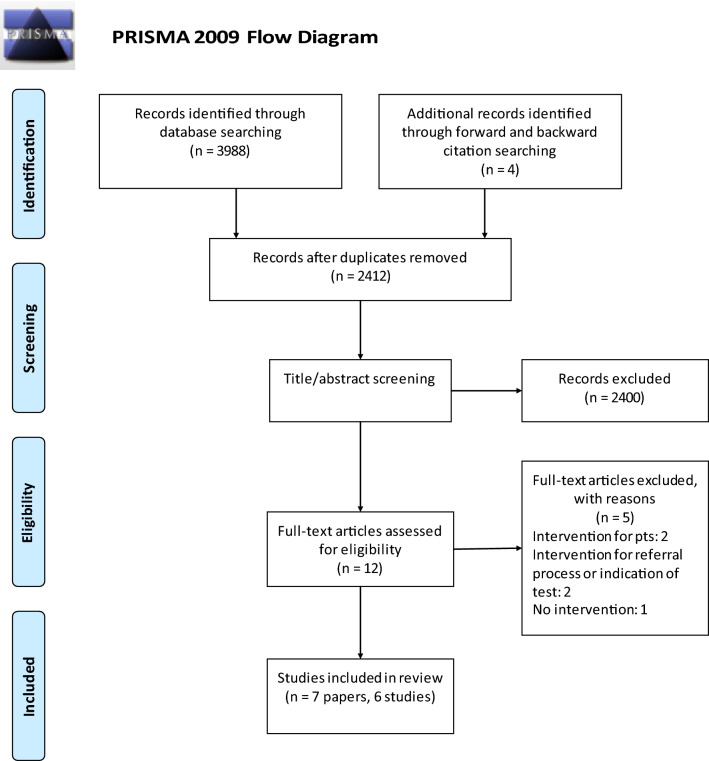
The PRISMA 2009 flow diagram

Once duplicates were removed, titles and abstracts were screened by two authors (RS, VS) for relevance. The remaining items were independently screened for their eligibility before confirming the final selection of papers for forward and backward searching. Those articles identified through forward/backward citation searching were confirmed against the eligibility criteria before being added to the final selection (Fig. 1).

### Analysis

Data were extracted by one author (RS) and reviewed both for accuracy and completeness by a second (VS). The type of data extracted captured basic information about the article, the population, participant retention, the intervention itself including methods used, and the overall results.

Quality assessment was carried out for each study using the Effective Public Health Practice Project Quality Assessment Tool for Quantitative Studies (EPHPP) [38]. This tool was selected for its use in public health, with less reliance on statistical outcomes. The assessment comprised six components (selection bias, study design, confounders, blinding, data collection method, and withdrawals/dropouts), which were scored as ‘weak’, ‘moderate’, or ‘strong’. Papers were given a global rating using the same scale. While the review outcomes focused on intervention content, having a quality assessment tool allowed for standardized comparisons to be made across papers. Two reviewers (RS/VS) completed these assessments independent of one another before discussing and confirming global ratings.

## Results

The initial search returned 3,988 items, of which 1,588 were duplicates. The remaining 2,400 papers were reviewed by title and abstract for relevance, with eight items then screened against eligibility criteria. Of these, three were found to be pertinent with a further four identified through subsequent forward and backward citation searching. Seven papers meeting the inclusion criteria were included with two linked publications, reporting on the same study but focusing on different outcomes [39, 40]. These will be referred to separately for the purposes of discussing methods and outcomes.

### Sample characteristics

The final seven papers involved a range of HCP audiences, including PCPs [39–41], nurses [42–44], physicians [42, 43], genetic counselors [42], and medical students [45]. Three (four papers) of the six interventions used a randomized study design [39–41, 44]. Table 1 summarizes the extracted data from each study.

**Table 1:** Intervention characteristics and outcomes

Author, Year, Country	Population	N	Objectives	Training Content	Outcomes	Key results
*Randomized*						
Bell, 2014^a^, USA	PCPs	3442 approached 155 enrolled 77 intervention 78 control 121 completed 60 intervention 61 control	To explore whether an educational intervention was more effective than a control at: -Improving risk assessment for hereditary BC -Discussing genetic testing/counseling including ELSI	*Intervention:* 6 h web-based curriculum covering: -Background of genetic testing -Risk assessment -Practice behaviors -Communication skills -Intervention employed clinical cases/tutorials on epidemiology and ELSI *Control* Carried out self-directed learning covering clinical genetics, ELSI, doctor–pt interaction and clinical reasoning	Outcomes collected approximately 1 month after training *SP visits* were conducted and analyzed using qualitative appraisal of topics and questions covered by the PCP	*Participant differences* No significant grp differences in demographics, years of experience or experience with inherited BC *Results:* Arms did not significantly differ on counseling behaviors, soliciting personal history, or discussing ELSI Intervention arm more likely to discuss benefits of counseling (37/60.7% control, 47/78.3% intervention, *p* 0.05), encourage SP to make decisions after genetic counseling (13/21.3% control, 23/38.3% intervention, *p* 0.05), ask about male relatives with prostate cancer (*p* 0.006), and discuss increased risk of cancer for male relatives (1/1.6% control, 12/ 20% intervention, *p* 0.001) Intervention grp significantly less likely to ask about Ashkenazi Jewish heritage (21/34.4% control, 8/13.3% intervention, (*p* 0.01) When asked “what would you do”, intervention grp significantly less likely to respond definitively that they would get tested (33/54.1% control, 20/33.3% intervention, *p* 0.03), and more likely to say it was the SP’s decision (n.s.)
Houwink, 2014, The Netherlands	GPs	2100 approached 88 enrolled 46 intervention 42 control 56 completed 38 intervention 18 control	*Aim of improving* -Recall of details about hereditary cancer -Recognition of pts at risk of inheritance -Pedigree drawing -Discussing hereditary risks, including management and ethical issues -Identification of pts for referral of risk assessment -Explaining the possibilities and limitations of oncogenetic testing -Knowing when to contact a genetic specialist	*Intervention* Training featured: -Hereditary forms of cancer -Discussion with pts who had a history of BRCA testing -Role-play of scenarios *Control:* Did not receive any additional training; this was offered later	All participants received a visit from an SP at baseline, 1, & 3 months post intervention *Intervention grp only* Questionnaires on applicability of gained knowledge and satisfaction with training. Latter questionnaire asked whether participants would recommend training and if it was relevant to practice	*Participant differences:* Grps did not differ significantly on age, sex, years, or type of experience or pre-test performance *Results* *SPs* Significant between grp performance difference of 0.19 (*p* < 0.0005) 1 month post intervention, not present at baseline or 3 months post Further regression analysis covarying for pre-test score, using the control grp score as reference, revealed a moderate effect size at 1 month post (standardized regression coefficient = 0.34) which was reduced, though still moderate, at 3 months (standardized regression coefficient = 0.28) *Applicability* Questionnaire completed by 17/38 participants; 65% reported applying their learnt skills monthly, 35% weekly *Satisfaction* Questionnaire completed by 18/38 Average response to both questions was 4.4/5 Average global grade for course was 7.7/10
Masny, 2008, USA	Nurses: 80% from a community setting 20% from a cancer center	Unk approached 41 enrolled 20 intervention 21 control 25 completed, unk by arm	Program aimed to improve: -Understanding of clinical cancer genetics -Knowledge of genetic assessment, syndromes and pedigrees -Communication of testing benefits and limitations -Evaluation of ELSI and psychosocial issues for individuals and their family -Integrate knowledge of risk estimation models -Development of follow up plans Apply skills to disclosure -Utilization of a mentor service to improve critical thinking and counseling skills	*Intervention* Training covered: -Review of molecular cancer genetics -Cancer risk assessment -Benefits and limitations of genetic technologies -Case studies illustrating psychosocial and ELSI concerns -Cancer risk estimation -Medical management for high-risk breast/ovarian carriers -Pre- and post-test counseling including decisional support -Overview of mentorship and professional development *Control* Same training but mentorship delayed 3 to 6 months from course completion	Mentorship program assessed on increased networking skills, self-efficacy and provided support for continued learning Changes to networking and self-efficacy assessed by questionnaire at baseline, 3, and 6 months Mentorship activity documented via a log with a telephone interview between participants and health educators to discuss the experience	*Participant differences* Grps did not differ on education, working with, or experience of, a Cancer Risk Assessment program *Results* Analyses are combined for control and intervention arms There was a significant improvement in self-efficacy for the entire grp including: explanation of genetic concepts, assessment of cancer risk, development of differential syndrome list, interpretation and disclosure of results, discussion of cancer screening, chemoprevention and prophylactic surgery (all *p* < 0.05 with an average mean change across domains of .91 on a scale of 1–5) There was an increase in networking between genetic nurses and counselors from 17 participants at baseline to 33 at 6 months Participants felt there was benefit to the mentoring program
Wilkes, 2017^a^, USA	See Bell 2014				*Self-efficacy* 28 items; 20 capturing self-efficacy for genetically related skills or knowledge, pre and post intervention, both grps *Knowledge* 43 items covering breast/ovarian cancer genetics, genetic testing, SDM, ELSI, venous thromboembolism, and perinatal/pediatric genetic testing. Pre and post intervention, both grps *Program assessment* Grps assessed curriculum on a number of features *Pre- and Post-intervention objective structured video exercises (OSVE)* 6 videos assessing clinical thinking, judgment, and intended behaviors *SP visit* This SP was a woman at risk for inherited BC visiting about 5 weeks after the intervention. This was recorded, transcribed, and coded	*Participant differences* No significant between grp differences for demographics, years of practice, or experience with inherited BC *Results:* 92% of intervention participants viewed all of the content, control participants an average of 87% *Self-efficacy* Clinical skills efficacy and genetic knowledge, both assessed on a scale of 1–5, improved in both arms *p* < 0.01, with significant between grp differences (p 0.02 for both). Clinical skills self-efficacy: Intervention: 3.0 (2.8 – 3.1)—> 3.8 (3.6–3.9) Δ = 0.8 (0.6–0.9); Control: 3.0 (2.9–3.2)—> 3.6 (3.5–3.7) Δ = 0.6 (0.4–0.7). Genetic knowledge: Intervention: 2.4 (2.3 – 2.6)—> 3.4 (3.3–3.6) Δ = 1.0 (0.9–1.1); Control: 2.5 (2.4—2.6)—> 3.3 (3.1–3.4) Δ = 0.8 (0.6–0.9) *Knowledge* Both arms significantly improved their cumulative knowledge, knowledge about BRCA genetics, BC, ELSI, and ethics in the pediatric setting, all reported as % correct (Average mean change for intervention: 17.50, for control: 8.25, *p* < .05 for all within and between grp comparisons aside from ethics in the pediatric setting—between grp *p* 0.08) Significant improvement in the intervention grp only was seen for knowledge about genetic testing (67.9—> 80.0, *p* < 0.01 for within [intervention only] and between) and general genetics/VTE (65.5—> 86.9, *p* < 0.01 for within [intervention only] and between) Neither grp improved in SDM *Program assessment* Intervention grp significantly more likely to rate the course highly and recommend to colleagues (*p* < .0001 for both, no further detail available) *OSVE* Both grps improved significantly (Intervention 11.4—> 14.2 *p* < 0.01; Control 11.2–> 13.8 *p* < 0.01 for both) without significant between grp differences. Possible scores ranged from 0–40 *SP visits* SPs tended to score the intervention arm more highly on their communication skills, offering options for care, being encouraged to ask questions, feeling listened to and having enough time Participants in general asked about 20% of the personal and family history questions but did not spend as much time on psychosocial concerns
*Not randomized, with a control*						
McGovern, 2010, USA	3rd year medical students	Unk approached 136 enrolled on SP program 121 completed 63 controls completed 6-month assessment. Unclear how many controls completed baseline assessment	To assess SP program at improving medical students’ assessment and communication of genetic information and risk	All students received an orientation lecture including information about cancer genetics, BRCA, surveillance options, risks and benefits of testing, ELSI, and pedigree analysis. A sub-set of these students took part in an SP program *Intervention* Students met with SP twice: pre-disclosure and disclosure Students were expected to capture a pedigree, medical history, discuss testing options, and deliver a result with explanation of its meaning and implications for treatment and family concerns	*Skill level* 5-point Likert scale to self-assess skill levels for: -taking medical and family histories -drawing a pedigree -assessing and communicating genetic risks All students completed this prior to orientation lecture and 6 months after the program. Students in the SP program also completed this prior to each visit *SP checklist* SPs completed a checklist about the student’s communication skills following each visit *Knowledge test* 8-item test given before the 2nd SP visit *Student evaluation* Students evaluated SP program after each visit on a 5-point Likert scale for clarity of instruction, realism, adequacy of time, usefulness of the exercise, and any areas to improve their own skills	*Participant differences* Not included *Results* *Skill level* On a 5-point scale, skill levels improved in the intervention grp between baseline and prior to SP1 on drawing a pedigree (1.6—> 3.6; *p* < 0.01) and assessing genetic risk (1.3–> 2.7; *p* < 0.05); and between prior to SP1 and prior to SP2 on obtaining medical history (3.3—> 4.1; *p* < 0.05), assessing genetic risk (2.7–> 4.3; *p* < 0.01), and communicating risk (2.4–> 3.8; *p* < 0.01) Significant differences in skill levels were reported between the intervention and control grps at 6 months on drawing a pedigree (4.2, 2.4; *p* < 0.01), assessing genetic risk (3.7, 1.9; *p* < 0.01), and communicating risk (3.9, 2.2; *p* < 0.05); however, this analysis is problematic as baseline scores for control grp are not reported *SP checklist* These assessments demonstrated that students had good contracting, communicating, and closure skills with 90% eliciting a family history at the 1st encounter Only 60% explored how the SP could use the results or provided any visual aids In the 2nd encounter, 96% were able to communicate medical risks and management options but only 60% could explain the risk of cancer or address emotional concerns *Student evaluation* 90% of participants agreed that the program was useful in identifying areas for improvement with 95% noting improved confidence
*No comparator*						
Blazer, 2005, USA	Genetic counselors, masters level advanced nurse practitioners, physicians	Unk approached 40 enrolled 29 completed	-Demonstrate knowledge of relevant genetic and oncology principles -Assess personal and family history for pedigree analysis -Recognize features of hereditary cancer syndromes -Apply risk assessment and testing strategies -Recommend risk-appropriate surveillance and prevention -Communicate ELSI and psychological issues -Participate in MDT of practitioners	Course covered basic genetic material including a wet lab, oncology content, cancer risk counseling skills with mock risk assessments and practice sessions, and hereditary cancer syndromes Participants attended a 2-week on-site training program Course was delivered using workshops, journal clubs, MDT attendance, wet labs, practice counseling sessions, and a cancer genetic conference	*Knowledge test* 40 true/false items pre and post course covering cancer genetics, oncology, cancer syndrome recognition, cancer risk assessment, genetic testing, and ELSI *Professional development and practice outcomes survey* A checklist of prescribed post-course activities was completed 6 months after course completion with a clinical practice survey completed after a year *Participant satisfaction* CME evaluation of each module was attached to the final exam	*Knowledge* There was a significant knowledge gain from 65 to 80% correct post intervention (t(39) = 6.67; *p* < .0.001) *Professional development* 35 (80%) of participants completed and returned this evaluation at 6 months. All respondents had completed their designated reading assignments and some presented the information to other professionals Of the 29 participants who responded to the 1-year post-course survey, 7 had, or were in the process, of setting up a new genetic risk assessment practice 22 (76%) said the course had improved their professional self-efficacy and 18 (62%) said the course improved their ability and confidence *Participant satisfaction* 95% of respondents said they continued to use and benefit from the course material
Lee, 2013, Korea	Doctors, nurses, and 1 ‘other medical professional’	Unk approached 35 enrolled 33 and 30 completed the final awareness and knowledge test, respectively	To assess the effect of genetic counseling education on HCP knowledge, awareness, and counseling skills for pts at high risk for HBOC	3-day training event with lectures on cancer genetics, hereditary BC, HBOC of the Korean population, genetic testing and interpretation, ELSI, and pre- and post-test counseling Hands on practice sessions for risk assessment, pedigree drawing, and counseling	Participants completed pre- and post-course 49 true/false knowledge questions along with questionnaires capturing: AW—awareness of genetic counseling education AK—confidence in ability to perform counseling AS—confidence in counseling practice	Overall knowledge scores improved significantly at post-test with 30 responders (37.40 ± 4.64—> 40.20 ± 2.52; maximum possible score 49, *p* = 0.002) with significant improvement in the areas of hereditary BC syndromes (5.58 ± 1.32—> 7.68 ± 0.77; *p* < 0.0001 9 items), pre-test counseling (10.0 ± 1.05–> 11.0 ± 0.98; *p* 0.001 12 items), and genetic testing & interpretation (3.20 ± 0.92–> 3.93 ± 0.25; *p* 0.001 4 items) Cancer genetics and post-test counseling were significantly worse (3.97 ± 1.16–> 3.20 ± 0.71; *p* 0.006 5 items and 9.66 ± 1.79–> 8.80 ± 1.45; *p* 0.02 12 items, respectively) For the 33 participants who completed the awareness and confidence questionnaires, there was significant improvement across all 3 categories (AW 2 items: 3.87 ± 0.55—> 4.72 ± 0.37 t 8.963, AK 7 items: 3.13 ± 0.87—> 4.35 ± 0.52 t 9.819, AS 9 items: 3.18 ± 0.76—> 4.27 ± 0.53 t 8.727, all *p* < 0.001) Of 12 participants who provided feedback, 6 identified the importance of communication skills. Most respondents wanted more educational programs about counseling practice

*ELSI* ethical, legal, and social issues, *GP* general practitioner, *PCP* primary care physician, *SDM* shared decision making, *SP* standardized patient

^a^Bell and Wilkes are linked papers reporting on the same study. The Bell paper focuses on the quantitative information stemming from the SP assessment, while Wilkes features self- and assessor-reported outcomes

### Intervention characteristics

One intervention utilized a web-based platform [39, 40], with the remaining five adopting face-to-face approaches [41–45] of traditional lecture formats [41–45], counseling role play scenarios [41–44], risk assessment practice [42–44], lab experience [42], patient discussions [41], and mentorship [44].

The training focused on topics such as communication/counseling skills [39–45], genetic testing principles [39, 40, 42–45], psychosocial and ethical, legal, and social issues (ELSI) [39, 40, 43–45], hereditary cancers/BRCA [41–43, 45], and risk assessment [39, 40, 44].

Interventions provided face-to-face were either completed in one day [41], over multiple days up to two weeks [42–44], or a longer expanse of time [45]. The web-based intervention took six hours to complete [39, 40].

### Outcomes

Across the papers, the most common outcome assessed was communication skills but there was a lack of specificity as to what this comprised [39–41, 43, 45]. There was an inconsistency as to whether communication outcomes referenced process work or the correct dissemination of information. For those studies that used standardized patients (SPs) to assess candidate performance, both elements of communication skills were referenced [39, 40, 45]. In one intervention, counseling skills were assessed before and after the training using a knowledge test, including items about the competences required during counseling and for disclosure of test results. Skills relating to counseling *prior* to genetic testing significantly improved, while those concerning test results significantly decreased post intervention [43]. While practice sessions were included within the intervention, there was an absence of information as to the specific communication elements imparted. Other studies captured communication skills via knowledge or efficacy measures, again without clear detail on content [42, 44].

Self-efficacy and confidence were reported as outcomes in four studies [40, 43–45]. This was sometimes described as confidence in counseling practice [43] or clinical skills efficacy [40]. In other studies, self-efficacy was broadened to include concepts such as assessing risk, drawing a pedigree, obtaining a medical history, interpreting results, and discussing screening [44, 45]. In general, self-efficacy scores significantly improved between pre and post intervention [40, 43–45]. Two papers with a comparator arm [40, 45] reported significant between group differences; however, only one reported post-intervention scores for both groups rendering it impossible to assess the true impact of the intervention [45].

Similarly, knowledge was assessed in four studies with tests covering topics such as genetic testing, shared decision making, ELSI, cancer genetics, and hereditary syndromes including breast cancer [40, 42, 43, 45]. One study compared knowledge scores pre and post attendance in both the intervention and control arms [40]. While neither arm improved on shared decision making, both had significant improvements in overall knowledge and subsets of BRCA genetics, breast cancer, and ELSI, with further significant improvements in the intervention arm for understanding genetic test ordering and general genetics. Two papers report significant gains in overall knowledge [42] [43]. One further study used a knowledge test but did not provide the scores within the current paper [45].

Four papers reported participant satisfaction with the training program [40–42, 45], often evaluated at the end of the program apart from one that assessed clarity of instruction, realism, and overall usefulness following each SP visit [45]. In another study, 95% of participants cited continued use and benefit from the course material [42]. A further paper noted that 12/35 attendees completed course feedback; most wanted more counseling practice with six individuals highlighting the importance of communication skills [43].

### Quality assessment

All studies were given a ‘weak’ global rating on the EPHPP (two or more of the six categories scored as ‘weak’), though studies did receive some ‘moderate’ and ‘strong’ scores in individual categories. No study outlined randomization procedures. Only one received a ‘moderate’ score for selection bias as there was enough information to assume the intervention group were similar to the target population [45]. Two studies reported group differences, or confounders, between the intervention and comparator at baseline [40, 41]. Another two described both the reliability and validity of their measures [40, 42], with reliability mentioned in a further two reports [39, 43] and validity by one other [41].

The quality assessment for drop-out and withdrawal rates presented a range of scores with three papers receiving a ‘weak’ rating due to a lack of transparency of baseline numbers or low completion rates [41, 42, 45], three a ‘moderate’ rating owing to the amount of drop outs [39, 40, 44], and one paper received a ‘strong’ rating [43].

## Discussion

This systematic review identified seven published papers featuring six interventions provided to HCPs communicating risk-based information about hereditary breast cancer. The participants were various groups of HCPs but primarily PCPs [39–41] and nurses [42–44]. The use of SPs was prominent across interventions to simulate that of a real clinical encounter. This provided participants with an opportunity to practice counseling skills, often resulting in improved self-reported efficacy and confidence. Within those studies that reported participant satisfaction, it was clear that individuals felt they had benefitted from their training and were keen to engage with more materials.

There was however a lack of detail about the specific communication behaviors included in training. From the descriptions provided, it was unclear how much the studies discussed the content of what to include in conversations versus the process of conveying that information. In line with genetic counseling models, which we used to inform our understanding of which communication skills were most likely to be included in training courses, we anticipated reference to both the correct dissemination of information alongside interpersonal skills such as empathic understanding and chunking and checking information [15, 20, 21, 23]. Successful genetic counseling conversations require not only an ability to explain risk in a manner that aids understanding but also rapport and empathy [15, 18]. Consultations should therefore incorporate components of genetic counseling, education, and psychosocial well-being [21]. While these topics may have been covered within the educational programs, they were not specifically referenced or reported. This review set out to understand the communication elements within HCP training programs, particularly the presence of both process and content-based skills. However, a lack of clear definition within the studies precluded a true assessment of their presence and impact.

Given the implications of genetic conversations, there is a need for interventions which address HCP confidence and skill when navigating these complex issues [5, 29, 31, 32]. This is especially true as more responsibility is given to a wider population of HCPs to engage in this dialogue. There was a dearth of reported interventions for oncologists or surgeons who may be the first point of contact for an individual. Previous systematic reviews have been conducted looking at groups of HCPs in isolation, such as PCPs [46]. However, as genetic consultations are being carried out in various ways, and often with different types of clinician working together (e.g., oncologist and geneticist), the focus in this review was on clinicians as a whole to reflect this changing landscape and a diverse MDT [23]. In addition, the lack of relevant published papers, and the overall weak quality scores, in this review indicates a need for robust evaluation of these training programs built on recommendations from the genetic counseling field.

### Research recommendations

A lack of definition across the studies for what constituted counseling skills demonstrates a need for future research to clearly operationalize this concept from the outset. There is a clear need for future training and assessment to focus on the process of communication [16, 19]. Our results suggest this could be beneficial as it was not clear from the interventions whether or not ‘good’ communication was characterized solely by the correct transmission of factual information or the actual process and engagement. The use of a framework or inclusion of specific communication tools would add clarity to what is currently an ambiguous understanding.

The psychosocial support provided to individuals during a genetic consultation is just as important as knowledge exchange [18, 20, 26] yet only two studies reported on a general lack of discussion between HCP and SP about these concerns [40, 45]. As these elements are a key feature of risk-based testing and decision making, it is important for future work to be more explicit as to how training targets the ability to communicate in this way.

While the client–HCP interaction is paramount, it is also necessary to explore interdisciplinary communication. This is especially so as more genetic testing information is provided via a team approach. While our findings demonstrate training materials are available, we did not identify any published interventions that were available for multi-disciplinary teams (MDTs). For example, genetic referral pathways may include surgical, nursing, and oncology input and yet training was not available in one setting, which can help ensure consistent communication. To that end, we have secured funding to develop a training program in this area, which will be informed by this systematic review.

### Practice implications

While the predominant outcomes of this review relate to future research, there are still practice implications to be gleaned. With a lack of published training targeting a complete MDT, it is important for colleagues to work together to understand how genetic information is being relayed to avoid confusion or contradiction.

This review suggests that some studies convey measurable benefits for HCPs, many of whom desire to undertake further counseling training. More evidence-based interventions may then assist HCPs when talking with individuals who potentially carry gene mutations.

### Study limitations

We identified only those papers published in peer-reviewed journals and there may be further information in gray literature and conference abstracts/editorials/letters. Our focus on published material was to understand the training programs which had been evaluated in some way. There may be other interventions available for HCPs that have gone unreported. In hindsight there may have been limitations to using the EPHPP tool. While this is a good quality assessment measure, the nature of the papers inherently leant themselves to receive lower scores primarily due to non-randomization and lack of control arms. However, we felt it was important to use a tool to help standardize our assessments without prior knowledge of the types of studies our searches would find.

## Conclusion

This systematic review set out to explore what published training interventions were available for HCPs discussing genetic testing and hereditary risk for breast cancer. This process demonstrated a lack of formally evaluated training programs. All seven papers reported on communication outcomes with particular use of an SP. Other outcomes such as knowledge, confidence/self-efficacy, and program satisfaction were captured. However, what is evident is a lack of consistent training materials used, with demonstrable paucity of support specifically for oncologists/surgeons.

There is increasing demand for genetic services within breast cancer, either to identify inherited risk or to personalize treatment options. In response to this, there is a commensurate need to ensure those HCPs tasked with helping individuals navigate the complex world of genetic breast cancer testing are well versed in conveying risk-based information.

## Electronic supplementary material

Below is the link to the electronic supplementary material.

Supplementary file1 (DOCX 15 kb)
