# Supplementary material for: A systematic review of communication interventions to help healthcare professionals discuss genetic testing for breast cancer
Source: Breast Cancer Res Treat. 2020 Jun 23;183(1):9–21. doi: 10.1007/s10549-020-05741-z (PMC7376076; doi:10.1007/s10549-020-05741-z)
Supplement: Supplementary file 1 — Supplementary file1 (DOCX 15 kb) [file 10549_2020_5741_MOESM1_ESM.docx]

|  | Term | Category |
| --- | --- | --- |
| 1 | Genetic counsel* ti,ab | Healthcare Professionals |
| 2 | Genetic* ti,ab |  |
| 3 | Oncologist ti,ab |  |
| 4 | Practitioner ti,ab |  |
| 5 | Physician ti,ab |  |
| 6 | Clinician ti,ab |  |
| 7 | Surgeon ti,ab |  |
| 8 | Or/1-7 |  |
| 9 | Communicat*  ti,ab | Communication |
| 10 | Inform*  ti,ab |  |
| 11 | Understanding ti,ab |  |
| 12 | Discuss* ti,ab |  |
| 13 | Or/9-12 |  |
| 14 | Interven*  ti,ab | Interventions |
| 15 | Train*  ti,ab |  |
| 16 | Teach*  ti,ab |  |
| 17 | Educat* ti,ab |  |
| 18 | Or/14-17 |  |
| 19 | "Breast Cancer"  ti,ab, kw | Population |
| 20 | "Breast Neoplasm"  ti,ab |  |
| 21 | 19 OR 20 |  |
| 22 | 8 AND 13 AND 18 AND 21 |  |

Supplement 1 – Search strategy
